# Resveratrol Alleviates Fumonisin B1-Induced Cytotoxicity in Sertoli Cells

**DOI:** 10.3390/foods13233810

**Published:** 2024-11-26

**Authors:** Song Yu, Lianpeng Zou, Jiawei Zhao, Yiping Zhu

**Affiliations:** Division of Chemical Toxicity and Safety Assessment, Shanghai Institutes of Preventive Medicine, Shanghai 200336, China; zoulianpeng@scdc.sh.cn (L.Z.); zhaojiawei@scdc.sh.cn (J.Z.); zhuyiping@scdc.sh.cn (Y.Z.)

**Keywords:** fumonisin, testicular toxicity, resveratrol, oxidative stress, toxicity intervention

## Abstract

Fumonisin B1 is a common food contaminant that has been found to adversely affect the reproductive system, especially Sertoli cells. However, the potential mitigation of FB1-induced cytotoxicity in Sertoli cells has not been fully elaborated. Resveratrol is a natural substance with anti-inflammatory, antioxidant, and anti-tumor properties. Herein, the protective effects of resveratrol against FB1-induced cytotoxicity in Sertoli cells were examined in this work. The mouse Sertoli cell line (TM4) was used as a research model. These results indicated that FB1 (40 μM and 80 μM) significantly reduces cell viability, disrupts the cell barrier, and induces an inflammatory response in TM4 cells. To our surprise, resveratrol (15 μM) showed an ability to reverse adverse effects induced by FB1 (40 μM). Furthermore, resveratrol could alleviate the FB1-induced apoptosis, decrease ROS level, and promote the antioxidant enzymes (CAT and SOD2) expression in FB1-treated TM4 cells. The addition of resveratrol could mitigate FB1-induced promoted phosphorylation of JNK and upregulation of c-jun expression. Interestingly, resveratrol was also able to mitigate the cytotoxicity of FB2 (40 μM), FB3 (40 μM), and an FB1-FB2-FB3 (40 μM-40 μM-40 μM) combination group on TM4 cells. In summary, this research displayed that resveratrol may alleviate fumonisin B1-induced cytotoxicity in Sertoli cells via inhibiting oxidative stress-mediated JNK/c-jun signaling pathway-induced apoptosis. This study provides new insights into the prevention and treatment of FB1-induced testicular toxicity and highlights the potential application value of resveratrol.

## 1. Introduction

Mycotoxins are secondary metabolites naturally produced by fungi. Mycotoxin contamination in cereals is to be higher than 25% annually, as estimated by the World Food and Agriculture Organization (FAO) [1]. What is more, they also pollute the environment by cleaning grain wastewater, domestic sewage, and rotting plants in the rivers [2]. Fumonisin B1 (FB1) is one of the common water-soluble mycotoxins produced mainly by *Fusarium proliferatum* and *Fusarium verticillioides*. It had been detected in water [2,3], feed [4], and food such as wheat, maize, rice, beer, and nuts [5]. In Pakistan, 90% of the 133 wheat samples were positive for FB1, 63% of which were above the EU limit of 200 μg/kg [6]. In Brazil, 82% of maize and maize products contained FB1, with an average of 0.398 mg/kg and a maximum of 3.46 mg/kg in 2010 [7]. Unfortunately, fumonisin B1 is capable of causing hepatotoxicity, nephrotoxicity, gastrointestinal toxicity, and immunotoxicity in animals and humans and is relevant to liver cancer, esophageal cancer, and neonatal neural tube defects [8,9]. The International Agency for Research on Cancer has classified it as a class 2B carcinogen [10].

The health of the male reproductive system is critical to human and animal reproduction. Previous studies have found that FB1 was able to reduce ejaculate volume, sperm concentration, and sperm quality in pigs and rabbits [11,12]. The testes, which are important for the production of androgens and the formation of sperm, are very sensitive to pollutants and can be easily impaired by them [13]. Sertoli cells are essential for maintaining spermatogenesis and testicular growth. Therefore, pollutant-induced cellular damage may lead to dysfunction of Sertoli cells, which may hinder spermatogenesis [14,15]. Some studies have found that FB1 promotes Sertoli cells’ oxidative stress, apoptosis, and cytostructural disruption [16,17]. However, there are few studies on mitigation methods for Sertoli cell injury caused by FB1.

Natural active substances have protective effects against the hazards caused by pollutants. Magnolol could ameliorate dysfunction of lipid metabolism induced by fumonisin B1 and oxidative damage in astrocyte-like C6 cells [18]. Melatonin reverses liver toxicity caused by ochratoxin A [19]. Myriocin alleviates damage to gastrointestinal cells and carcinogenesis of esophageal epithelial cells [20]. Resveratrol is a plant extract with antioxidant, anti-inflammatory, and anti-tumor properties [21]. It demonstrated that resveratrol could relieve the toxicity of mycotoxins, such as zearalenone, deoxynivalenol, and beauvericin [22,23,24]. However, the protective effect of resveratrol against FB1-induced Sertoli cell damage remains unclear. Therefore, our study explored the protective effects of resveratrol on FB1-induced cytotoxicity and its potential molecular mechanisms in Sertoli cells. With these results, a new method to alleviate FB1 toxicity on testis is provided for humans and animals.

## 2. Materials and Methods

### 2.1. Reagents

Fumonisin B1 (ab142433) and fumonisin B2 (ab142434) were from Abcam (Cambridge, MA, USA). Fumonisin B3 (20434) was purchased from Cayman (Ann Arbor, MA, USA). Fetal bovine serum (10099141C), horse serum (16050122), TRIzol reagent (15596018), and DMEM-F12 medium (10565018) were obtained from Gibco (Waltham, MA, USA). Direct-zol RNA MiniPrep (BJR205-D-200) was from Bojing Biotechnology (Shanghai, China). Penicillin–streptomycin antibiotics (03-033-1B) and 0.25% trypsin (with EDTA) (03-050-1B) were purchased from BioInd (Kibbutz Beit, Israel). The apoptosis assay kit (556547) was produced by BD Pharmingen (San Diego, CA, USA). Cell counting kit-8 (CCK-8) (AW-G9811) was purchased from Shanghai Aoyuan Biotechnology (Shanghai, China). The lactic dehydrogenase (LDH) assay kit (CK12-2000T) was from DOJINDO Laboratories (Kumamoto, Japan). Blocking buffer (P0252), reactive oxygen species (ROS) level assay kit (S0033M), protein concentration kit (P0010), and nitrocellulose film (FFN02) were from Beyotime Biotech (Nantong, China). All antibodies (Bax (2772S), caspase-3 (9668S), c-jun (9165S), Bcl2 (15071S), actin (3700S), phospho-SAPK/JNK (4668S), and secondary antibody (7074S)) were from Cell Signaling Technology (Danvers, MA, USA). Resveratrol (RET) (R107315) was purchased from Shanghai Aladdin Biochemical Technology (Shanghai, China). SYBR dye (RR820A) and PrimeScript^TM^ RT Master Mix (RR036A) were from Takara Biomedical Technology (Beijing, China).

### 2.2. Cell Culture

The mouse Sertoli cells (TM4) were obtained from Shanghai Aoyuan Biotechnology (Shanghai, China). TM4 cells were cultured in F12 medium (5% fetal bovine serum, 2% penicillin–streptomycin solution, and 5% horse serum) and placed at 37 °C in a 5% CO_2_ environment. When the cell density reached 60–70%, the cells were treated or passaged according to 1:2. The concentration was 0, 10, 20, 40, and 80 μM in the fumonisins alone group; the concentration of each fumonisin (FB1/FB2/FB3) was 40 μM in the FB1 + FB2 + FB3 group. Resveratrol has treated cells at concentrations ranging from 0, 5, 10, 15, and 20 μM. In the fumonisins-resveratrol group, the cells were pretreated with the resveratrol (15 μM) for 24 h, then were given 40 μM fumonisins.

### 2.3. Cell Proliferation Assay

After the TM4 cells were cultured in a 96-well plate for 24 h, the medium was aspirated, and then 100 μL of medium containing fumonisins or resveratrol was added according to Section 2.2. After 24 h or 48 h of incubation, 10 μL solution from cell counting kit-8 (CCK-8) (Shanghai Aoyuan Biotechnology) was added to each well. Then, it was incubated at 37 °C for 2 h. The absorbance at 450 nm was detected by applying an automated microplate reader (Thermo Fisher Scientific, Waltham, MA, USA).

### 2.4. Reactive Oxygen Species Level Assay

After the TM4 cells were cultured in 6-well plates for 24 h, the cells were treated with fumonisins or resveratrol according to Section 2.2. After 48 h, the TM4 cells were incubated with 10 μM 2′,7′-dichlorofluorescein diacetate (Beyotime Biotech) for 30 min by the manufacturer’s instructions of the reactive oxygen species (ROS) assay kit (Beyotime Biotech). The fluorescence intensity was recorded at an excitation wavelength of 480 nm and an emission wavelength of 525 nm using an automated microplate reader (Thermo Fisher Scientific, Waltham, MA, USA).

### 2.5. Lactate Dehydrogenase Detection Assay

After the TM4 cells were cultured in 96-well plates for 24 h, the cultured medium was aspirated, and 100 μL of medium containing fumonisins were added according to Section 2.2. After 24 h or 48 h of incubation, the positive control group was incubated with 10 μL of lysis buffer (DOJINDO Laboratories) for 30 min. Then, 100 μL of working solution (DOJINDO Laboratories) was added to each well and incubated for 20 min at room temperature in the dark, followed by 50 μL termination solution (DOJINDO Laboratories). The absorbance at 490 nm was detected by applying an automated microplate reader (Thermo Fisher Scientific, Waltham, MA, USA).

### 2.6. Cell Apoptosis Assay

After the TM4 cells were cultured in 6-well plates for 24 h, the TM4 cells were incubated with fumonisins or resveratrol per Section 2.2 for 48 h. The TM4 cells were digested by 0.25% trypsin for 2 min and harvested by centrifugation for 5 min (1000 rpm). The TM4 cells were washed once by phosphate buffer solution (PBS). Then, 90 μL buffer, 5 μL propidium iodide, and 5 μL AnnexinV-FITC (BD Pharmingen) were added. After incubating in a dark room for 15 min, the apoptosis rate was detected by flow cytometry (Beckman, Brea, CA, USA).

### 2.7. Real-Time Quantitative PCR Analysis Assay

The TM4 cells in 6-well plates were incubated with fumonisins or resveratrol according to Section 2.2 for 48 h. The cells were washed with PBS. Total RNA was harvested by TRIzol reagent and Direct-zol^TM^ RNA MiniPrep kit in cells. cDNA was synthesized using the extracted RNA as a template and the PrimeScript^TM^ RT Master Mix kit at 37 °C for 15 min. The gene expression level was quantitatively analyzed by SYBR Premix Ex Taq II reagent. The gene expression levels were analyzed using the CT comparison method. The primer sequence information is shown in Table S1.

### 2.8. Immunoblotting Assay

The TM4 cells in a 60 mm cell culture dish were incubated with fumonisins or resveratrol according to Section 2.2 for 48 h. The TM4 cells were washed once with PBS, and 150 μL of RIPA protein lysate (containing protease and phosphatase inhibitors) was added to a 60 mm dish. The cells were lysed for 30 min once (vortexing at 10 min intervals) before being centrifuged for 20 min at 13,000 rpm and 4 °C to harvest protein. The protein level was detected by the Protein Quantification kit. A protein sample was mixed thoroughly with 5X loading buffer and heated for 10 min at 100 °C. An equal volume of protein samples (20 μg protein) was incorporated into SDS-polyacrylamide gel electrophoresis, which was run for 15 min at a constant pressure of 80 V and then at 100 V until the end of the experiment. Then, the proteins were transferred to nitrocellulose film by running for 120 min at 300 mA constant current. The nitrocellulose film was sealed with blocking buffer on a decolorizing shaker at room temperature for 20 min and washed 3 times with TBST rinse solution for 5 min per time. The primary antibody (dilution ratio: 1:2000) was incubated overnight at 4 °C and washed 3 times with TBST rinse solution for 10 min per time. After these, the secondary antibody was incubated for 120 min at room temperature and washed 3 times with TBST rinse solution for 10 min per time. The protein expression level was detected by a chemiluminescence detection system, and the protein band was analyzed by Image J software, 1.54k 15 September 2024 (Madison, WI, USA).

### 2.9. Statistical Analysis

All experiments were independently repeated three times, and GraphPad Prism 9 software (GraphPad Software Inc., San Diego, CA, USA) was used to analyze the data for significant differences. The *p* < 0.05 implied a significant difference compared with the control group.

## 3. Results

### 3.1. Mitigation Effects of Resveratrol on Cell Proliferation Inhibited by FB1 in TM4 Cells

**Figure 1:**
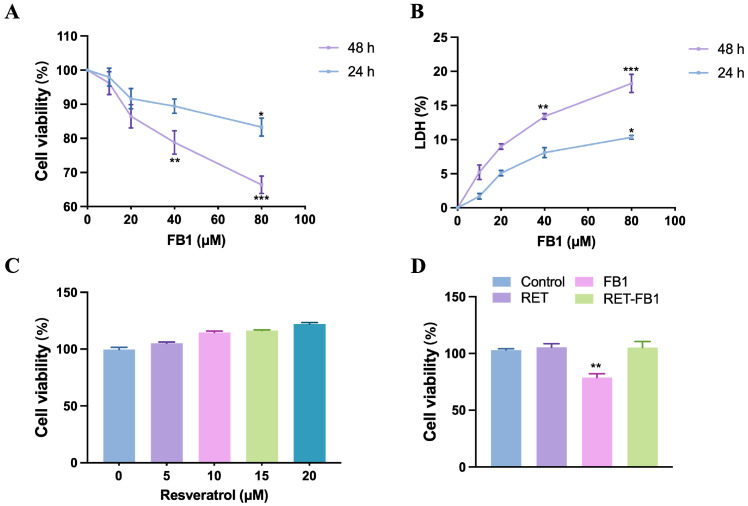
Resveratrol reversed FB1-induced cell proliferation suppression in TM4 cells. Cell viability (**A**) and lactate dehydrogenase level (**B**) were analyzed by cell counting kit-8 and lactate dehydrogenase kit after FB1 treatment (0, 10, 20, 40, and 80 μM) for 24 h or 48 h. (**C**) The effect of resveratrol (0, 5, 10, 15, and 20 μM) on cell proliferation at 48 h. (**D**) The effect of resveratrol (15 μM) on FB1 (40 μM)-induced cell proliferation suppression in TM4 cells. The data showed as the mean ± SD (* *p* < 0.05, ** *p* < 0.01, and *** *p* < 0.001).

The TM4 cells were treated with 0, 10, 20, 40, and 80 μM FB1 for 24 h or 48 h. As seen in Figure 1A,B, FB1 significantly increased lactate dehydrogenase (LDH) levels and decreased cell viability in a time- and dose-dependent manner (Figure 1A,B). After 48 h, the cell proliferation inhibition rate reached the highest level (36.9%) in the 80 μM FB1 group. Then, the alleviating effects of the resveratrol on FB1-induced adverse effects were evaluated. Resveratrol alone could not cause significant changes in TM4 cell viability (Figure 1C). The resveratrol (15 μM) was applied in follow-up experiments. With resveratrol, FB1 (40 μM)-induced inhibition of cell proliferation was alleviated (Figure 1D).

### 3.2. Protection Effects of Resveratrol on Cell Barrier Disruption and Inflammation Response Induced by FB1 in TM4 Cells

**Figure 2:**
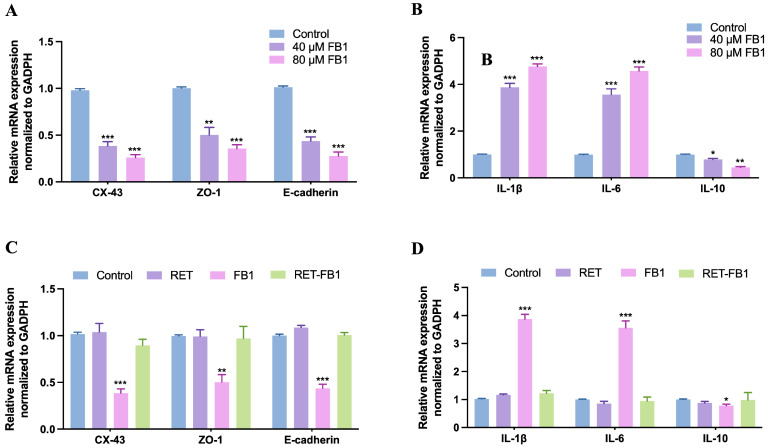
Resveratrol alleviated FB1-induced cell barrier damage and inflammatory response in TM4 cells. The mRNA expression levels of cell barrier protein (**A**) and inflammatory factor (**B**) were detected by real-time quantitative PCR analysis after FB1 treatment (40 μM and 80 μM) for 48 h. The effects of resveratrol (15 μM) on FB1-induced (40 μM) cell barrier damage (**C**) and inflammatory response (**D**) in TM4 cells. The data showed as the mean ± SD (* *p* < 0.05, ** *p* < 0.01, and *** *p* < 0.001).

The Sertoli cell barrier, which is a barrier against foreign substances in the testis, was mainly composed of adhesin junctions (E-cadherin) and tight junctions (Zonula occludens-1, ZO-1 and Connexin 43, CX-43) between Sertoli cells. The mRNA expression levels of tight and adhesins junction proteins (CX-43, E-cadherin, and ZO-1) were obviously decreased when exposed to 40 μM and 80 μM FB1 for 48 h (Figure 2A). Furthermore, the mRNA expression levels of pro-inflammatory factors (IL-6 and IL-1β) were markedly enhanced. In contrast, the mRNA expression level of IL-10, an anti-inflammatory factor, was significantly reduced (Figure 2B). This suggested that FB1 might disrupt the cell barrier and induce an inflammatory response in TM4 cells. However, there were no significant differences in the levels of cell barrier proteins and inflammatory factors in the RET (15 μM)-FB1 (40 μM) group compared with the control group (Figure 2C,D). The results implied that resveratrol could mitigate FB1-caused cytotoxicity in TM4 cells.

### 3.3. Resveratrol Attenuated FB1-Induced Apoptosis in TM4 Cells

**Figure 3:**
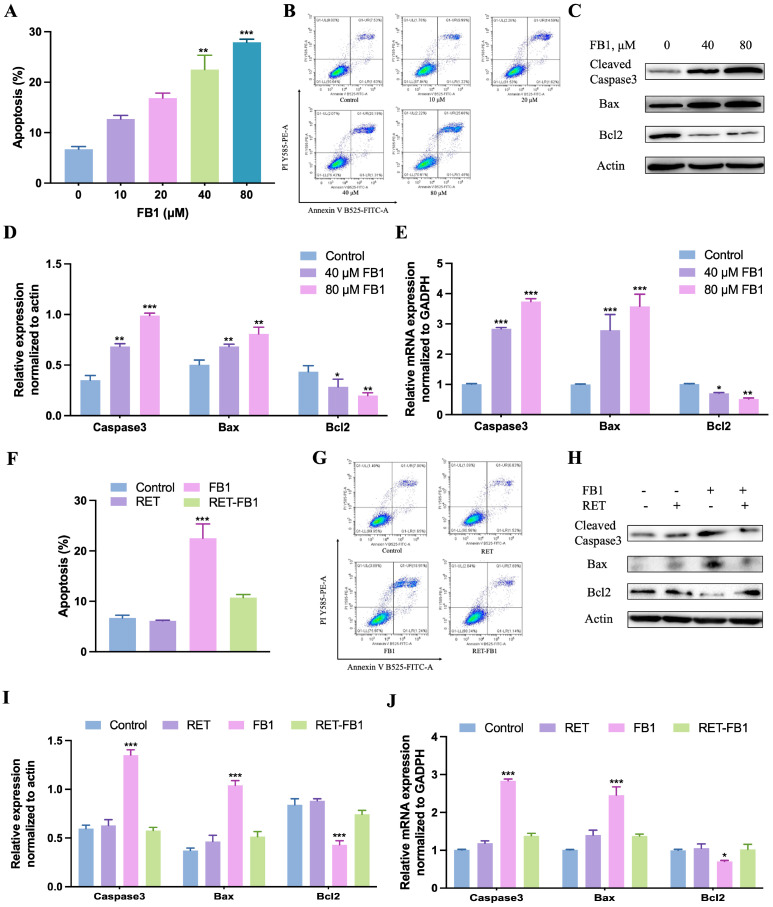
Resveratrol alleviated FB1-induced apoptosis in TM4 cells. (**A**,**B**) After treating TM4 cells with FB1 (0, 10, 20, 40, and 80 μM) for 48 h, the apoptosis rate was analyzed by flow cytometry. The expression levels of apoptosis-related genes were detected by immunoblotting (**C**,**D**) and real-time quantitative PCR analysis (**E**) after FB1 treatment for 48 h. (**F**–**J**) The effects of resveratrol (15 μM) on FB1 (40 μM)-induced apoptosis in TM4 cells. The quantification of the protein band was analyzed by Image J software. The data showed as the mean ± SD (* *p* < 0.05, ** *p* < 0.01, and *** *p* < 0.001).

Apoptosis is a common event in contaminant-induced cell injury. In this study, FB1-induced apoptosis was studied by flow cytometry, immunoblotting and real-time quantitative PCR analysis. As shown in Figure 3A,B, FB1 significantly increases the apoptosis rate in TM4 cells (Figure 3A,B). After FB1 was exposed for 48 h, the expression levels of pro-apoptotic genes (Caspase3 and Bax) were dramatically increased, but the level of anti-apoptotic gene (Bcl2) was notably reduced (Figure 3C–E). Resveratrol decreased the apoptosis rate (Figure 3F,G). When resveratrol was added, there were no significant differences in the expression levels of apoptotic genes compared to controls (Figure 3H–J). These data revealed that resveratrol mitigated FB1-induced apoptosis in TM4 cells.

### 3.4. Resveratrol Prevented FB1-Induced Oxidative Stress in TM4 Cells

**Figure 4:**
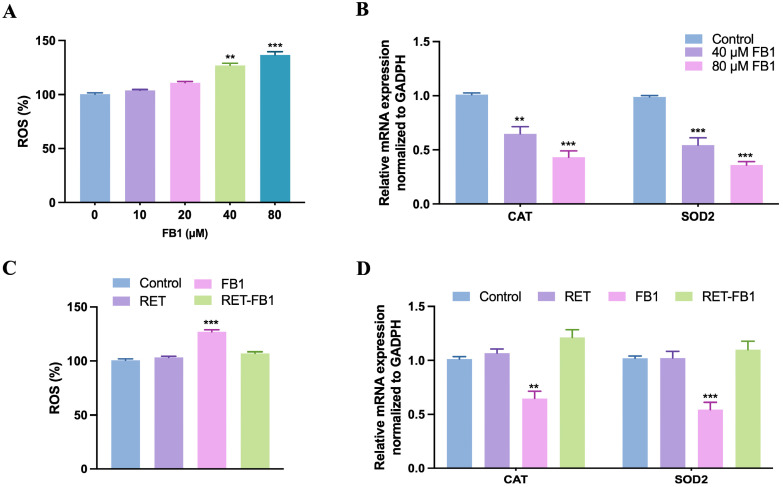
Resveratrol prevented FB1-induced oxidative stress in TM4 cells. The reactive oxygen species (ROS) were detected (**A**) by the ROS Assay Kit, and the mRNA expression levels of antioxidant enzymes were analyzed (**B**) by real-time quantitative PCR after FB1 treatment (0, 10, 20, 40 and 80 μM) for 48 h. (**C**,**D**) The effects of resveratrol (15 μM) on FB1 (40 μM)-induced oxidative stress in TM4 cells. The data showed as the mean ± SD (** *p* < 0.01 and *** *p* < 0.001).

The ROS level was markedly increased, and the mRNA expression levels of antioxidant enzymes superoxide dismutase 2 (SOD2) and catalase (CAT) were markedly decreased when FB1 treatment for 48 h (Figure 4A,B). We also found that resveratrol was able to reduce the ROS accumulation (Figure 4C) and increase the expression level of antioxidant enzymes (Figure 4D). These results suggested that resveratrol treatment prevented oxidative stress in FB1-treatment cells.

### 3.5. Resveratrol Inhibited FB1-Activated the JNK/c-Jun Signaling Pathway in TM4 Cells

**Figure 5:**
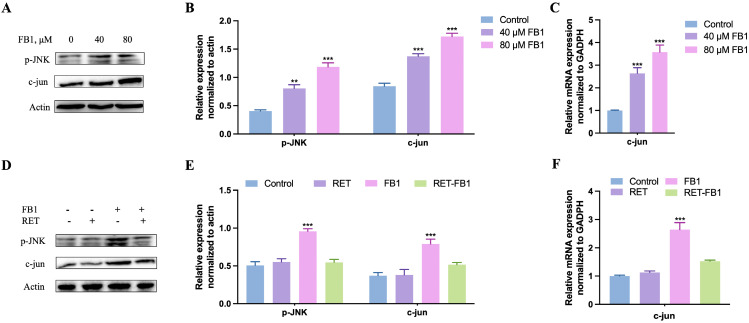
Resveratrol inhibited FB1-activated the JNK/c-jun signaling pathway in TM4 cells. Activation of the JNK/c-jun signaling pathway was detected by immunoblotting (**A**,**B**) and real-time quantitative PCR analysis (**C**) after FB1 treatment (40 μM and 80 μM) for 48 h. (**D**–**F**) The effects of resveratrol (15 μM) on FB1 (40 μM) activated the JNK/c-jun signaling pathway in TM4 cells. The quantification of the protein band was performed by Image J software. The data showed as the mean ± SD (** *p* < 0.01 and *** *p* < 0.001).

Previous research has shown that the JNK signaling pathway always participates in mycotoxin-induced toxicity as a downstream pathway of oxidative stress [25]. Our results showed that FB1 was able to promote JNK phosphorylation and enhance c-jun expression levels (Figure 5A–C). Surprisingly, in the RET + FB1 group, the levels of phosphorylation JNK and c-jun were not significantly different compared with the control group (Figure 5D–F). Taken together, resveratrol might alleviate FB1-induced cytotoxicity by blocking the ROS/JNK/c-jun signaling pathway-induced apoptosis.

### 3.6. Resveratrol Mitigated Fumonisin Combination-Induced Cytotoxicity in TM4 Cells

**Figure 6:**
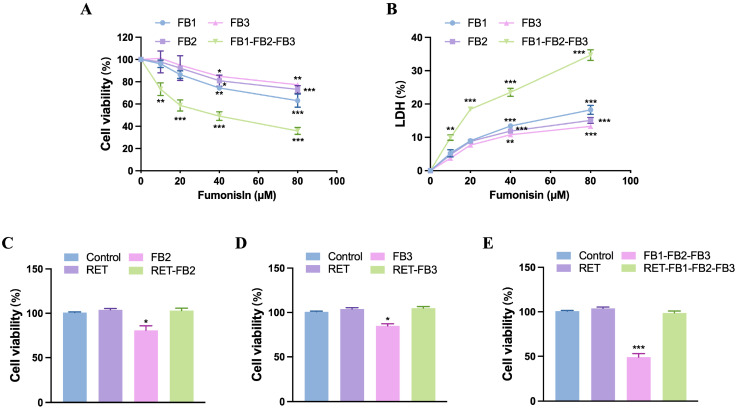
Resveratrol mitigated the cytotoxicity of the fumonisin combination in TM4 cells. Cell viability (**A**) and lactate dehydrogenase level (**B**) were analyzed by cell counting kit-8 and lactate dehydrogenase kit after fumonisin (0, 10, 20, 40, and 80 μM) treatment for 24 h and 48 h. (**C**–**E**) The effect of resveratrol (15 μM) on fumonisin (40 μM)-induced cell proliferation suppression in TM4 cells. The data showed as the mean ± SD (* *p* < 0.05, ** *p* < 0.01, and *** *p* < 0.001).

Fumonisin B2 (FB2) and Fumonisin B3 (FB3) are often present in feed and food together with FB1. This study is the first to investigate the cytotoxicity of FB2, FB3, and co-occurring three fumonisins in TM4 cells. As shown in Figure 6, a significant reduction in cell viability (Figure 6A) and a significant increase in LDH level (Figure 6B) were observed after FB2 and FB3-treated cells for 48 h, suggesting that FB2 and FB3 could induce Sertoli cell damage. The degree of cytotoxicity was significantly enhanced when three fumonisins were given simultaneously compared to mono treatment (Figure 6A,B), which also implied a synergistic effect of toxicity from the combined exposure of the three toxins. Resveratrol not only mitigated the cytotoxicity of FB2 and FB3 but also had a protective effect against the combined toxicity of FB1-FB2-FB3 (Figure 6C–E).

## 4. Discussion

Fumonisin B1 is one of the most prevalent mycotoxins and is found in corn and water [5]. To date, FB1 has been found in the liver, kidneys, and intestines. It causes pulmonary edema, cerebral leukoplakia, immunotoxicity, hepatotoxicity, nephrotoxicity, and carcinogenicity in humans and animals [9,26,27]. In recent years, a correlation between FB1 and reproductive disorders has been identified, with FB1 being able to reduce ejaculate volume and sperm motility in pigs and rabbits [11,12]. The testis, as an important male reproductive organ, provides nutrition and a stable microenvironment for sperm formation [14]. Sertoli cells, the major constitutive cells in the testis that provide nutritional and structural support for spermatogenesis, were major target cells of pollutant-induced testicular injury. Several studies have identified Sertoli cell damage by FB1 [16,17]. Currently, there are few studies on mitigation methods for Sertoli cell toxicity of FB1.

Given the mechanism of toxicity, the utilization of functional natural products to mitigate the toxicity of pollutants is regarded as a safe and efficacious strategy for toxicity intervention [19,28,29]. Resveratrol, an extract from plants such as grapes, tiger nuts, peanuts, and cassia, has anti-tumor, antioxidant, and anti-inflammatory activities. Resveratrol mitigates pollutant-induced intestinal, liver, and neurotoxicity in vivo and in vitro experiments [30,31]. Additionally, resveratrol has a mitigating effect on testicular damage. It significantly alleviated busulfan-induced changes in the reproductive system of rats [32]. 3-Monochloropropane-1,2-diol (3-MCPD) induced sterility in male rats, reduced testicular weight, and inhibited spermatogenesis; resveratrol relieved the effects of 3-MCPD on testicular weight and spermatogenesis in rats. Inflammation and disruption of steroidogenesis induced by 3-MCPD were also alleviated by resveratrol [33]. However, the effect of fumonisin B1 on Sertoli cells was not proved before this experiment. Our study was the first time that we evaluated the protective effect of resveratrol on FB1-induced injury in Sertoli cells. We found that resveratrol not only mitigated the cytotoxicity of FB1 but also FB2, FB3, and the combination of FB1-FB2-FB3 in Sertoli cells.

The structural integrity and viability of the Sertoli cells are essential for the maintenance of male reproductive function. The blood–testis barrier (BTB) is formed between the Sertoli cells and serves as an important structure in the testis. It maintains a stable microenvironment for spermatogenesis [34]. AFB1 and T-2 have been found to disrupt the integrity of the BTB and induce an inflammatory response as well [35,36]. These adverse effects were also found in the present study. FB1 decreased cell viability and increased cell membrane permeability in TM4 cells. And the expression levels of adhesin junctions (E-cadherin) and tight junctions (ZO-1 and CX-43) were compromised by FB1. The levels of IL-6 and IL-1β were notably increased in TM4 cells, whereas the level of anti-inflammatory factor IL-10 was markedly reduced after FB1 exposure. These results suggested that FB1 was capable of disrupting the structure and function of TM4 cells. Dysfunction of Sertoli cells may lead to impairment of sperm formation and premature release, which likely contribute to male infertility caused by FB1. Surprisingly, resveratrol was able to alleviate FB1-induced cell proliferation inhibition, cell barrier disruption, and inflammatory responses in this study. This is consistent with the protective effect of quercetin against FB1-induced cytotoxicity [37].

Apoptosis is always an early event in pollutant-induced cell injury, and Sertoli cell apoptosis could lead to testicular injury, such as disruption of BTB integrity and inflammatory responses [38]. FB1 increased the apoptosis rate and induced aberrant expression of apoptosis-related genes in TM4 cells. This is similar to the toxicity profiles of other mycotoxins in Sertoli cells [39]. Resveratrol relieved apoptosis induced by DON, T-2, and aflatoxin B1 in liver and intestinal epithelial cells [40]. Our results showed that resveratrol significantly decreased the apoptosis rate and the expression level of pro-apoptotic markers in TM4 cells.

Oxidative stress is a form of stress caused by drastic changes in the intra- and extracellular environment that break the redox balance [38]. It damages intracellular proteins, DNA, and lipids and leads to apoptosis by regulating intracellular signaling pathways such as PI3K/Akt and MAPK [38]. Oxidative stress has been found to be associated with cancer, cardiovascular disease, and neurological disease [41]. The relationship between oxidative stress and Sertoli cell damage has been demonstrated as well [42]. The blood–testis barrier was damaged by glyphosate via the NOX1-mediated generation of oxidative stress [43]. The furan compound induced reproductive toxicity by modulating oxidative stress, apoptosis, and inflammation in the testicular tissue [44]. Quercetin restores FB1-induced impairment of Sertoli cell proliferation and function by inhibiting ROS accumulation and enhancing the glycolysis process [37]. Similarly, our data demonstrated that FB1 increased ROS levels in TM4 cells and inhibited the expression of antioxidant enzyme genes. The JNK/c-jun signaling pathway often acts as a downstream of oxidative stress involved in pollutant-induced toxicity [45,46]. This pathway was activated by FB1 in TM4 cells in this study, with increased levels of JNK phosphorylation and c-jun expression. Also, resveratrol inhibited diabetes-induced oxidative stress and the JNK pathway in the testis and alleviated diabetes-induced testicular dysfunction [47]. This research demonstrated that resveratrol inhibited ROS accumulation and enhanced the expression of antioxidant enzymes. In addition, the FB1-activated JNK/c-jun signaling pathway was also inhibited by resveratrol. These results suggest that resveratrol may alleviate FB1-induced cytotoxicity by inhibiting ROS/JNK/c-jun-induced apoptosis in Sertoli cells.

Although fumonisin B2 (FB2) and fumonisin B3 (FB3) are usually found in feed and food with the existence of FB1, the toxicity of FB2 and FB3 to Sertoli cells has not been reported. These results suggested that FB2 and FB3 can decrease cell viability and increase LDH levels. Furthermore, together FB1, FB2, and FB3 had synergistically toxic effects. Resveratrol also mitigated cytotoxicity in the FB2, FB3, and FB1-FB2-FB3 combinations. This implied that resveratrol may be antidoted to prevent testicular toxicity of fumonisin. In the future, animal models will be applied to further confirm the protective effect of resveratrol against FB1-induced testicular toxicity and effective dose.

## 5. Conclusions

The protective effects of resveratrol against FB1-induced toxicity in Sertoli cells are first reported in this study. FB1-induced cell barrier dysfunction, inflammatory response, and cell proliferation inhibition. However, with resveratrol, the adverse effects caused by FB1 were mitigated. In addition, resveratrol inhibited oxidative stress and activation of the JNK/c-jun signaling pathway and prevented FB1-induced apoptosis. This study suggested the protective effect of resveratrol against FB1-induced damage in Sertoli cells. Surprisingly, resveratrol had similar protective effects on other fumonisins. This new finding is important for understanding the mechanism of FB1-induced Sertoli cell damage and potential toxicity intervention strategies. It is essential to protect animals and humans from the adverse effects of mycotoxin contamination on the reproductive system.

## Data Availability

The original contributions presented in the study are included in the article/Supplementary Material, further inquiries can be directed to the corresponding author.

## Supplementary Materials

The following supporting information can be downloaded at: https://www.mdpi.com/article/10.3390/foods13233810/s1, Table S1. RT-PCR primer sequence in testis sertoli cells.

## Abbreviations

FB1	Fumonisin B1
BTB	Blood–testis barrier
JNK	C-Jun N-terminal protein kinases
CCK-8	Cell Counting Kit-8
LDH	Lactic dehydrogenase
CAT	Catalase
SOD2	Superoxide Dismutase 2
RET	Resveratrol
TM4	Testicular Sertoli cells
ROS	Reactive oxygen species
ZO-1	Zonula occludens-1
CX-43	Connexin 43
IL-6	Interleukin 6
IL-10	Interleukin 10
3-MCPD	3-Monochloropropane-1,2-diol
