# Supplementary material for: Resveratrol Alleviates Fumonisin B1-Induced Cytotoxicity in Sertoli Cells
Source: Foods. 2024 Nov 26;13(23):3810. doi: 10.3390/foods13233810 (PMC11640369; doi:10.3390/foods13233810)
Supplement: Supplementary file 1 [file foods-13-03810-s001.zip › foods-3290485-supplementary.pdf]

Resveratrol Alleviates Fumonisin B1-Induced Cytotoxicity in Sertoli Cells

Song Yu *, Lianpeng Zou, Jiawei Zhao and Yiping Zhu

Division of Chemical Toxicity and Safety Assessment, Shanghai Institutes of Preventive Medicine, Shanghai 200336, China
* Correspondence: yusong@scdc.sh.cn; Tel.: +86-21-62758710

Table S1. RT-PCR primer sequence in testis sertoli cells.

Gene name	Primer sequence
Caspase3	F: ATGGAGAACAACAAAACCTCAGT<br>R: CGACCCGTCCTTTGAATTTCT
Bcl2	F: GCTACCGTCGTGACTTCGC<br>R: CCCCACCGAACTCAAAGAAGG
Bax	F: AGACAGGGGCCTTTTTGCTAC<br>R: AATTCGCCGGAGACACTCG
SOD2	F: CAGACCTGCCTTACGACTATGG<br>R: CTCGGTGGCGTTGAGATTGTT
CAT	F: AGCGACCAGATGAAGCAGTG<br>R: TCCGCTCTCTGTCAAAGTGTG
ZO-1	F: GCCGCTAAGAGCACAGCAA<br>R: TCCCCACTCTGAAAATGAGGA
CX-43	F: ACAGCGGTTGAGTCAGCTTG<br>R: GAGAGATGGGGAAGGACTTGT
E-cadherin	F: TGCTCCTACTGTTTCTACG<br>R: CTTCTCCACCTCCCTCTT
IL-1β	F: ACGGACCCCAAAAGATGAAG<br>R: TTCTCCACAGCCACAATGAG
IL-6	F: TAGTCCTTCCTACCCCAATTTCC<br>R: TTGGTCCTTAGCCACTCCTTC
IL-10	F: GCTCTTACTGACTGGCATGAG<br>R: CGCAGCTCTAGGAGCATGTG
c-jun	F: GAAGTGACGGACCGTTCTATGGAC<br>R: CGTTGAGGGCATCGTCGTA
GADPH	F: AAGAAGGTGGTGAAGCAGGCATC<br>R: CGGCATCGAAGGTGGAAGAGTG
